# Occurrence of Indicator Genes of Antimicrobial Resistance Contamination in the English Channel and North Sea Sectors and Interactions With Environmental Variables

**DOI:** 10.3389/fmicb.2022.883081

**Published:** 2022-05-16

**Authors:** Erwan Bourdonnais, Darina Colcanap, Cédric Le Bris, Thomas Brauge, Graziella Midelet

**Affiliations:** ^1^ANSES, Laboratoire de Sécurité des Aliments, Unité Bactériologie et Parasitologie des Produits de la Pêche et de l’Aquaculture, Boulogne-sur-Mer, France; ^2^Univ. du Littoral Côte d'Opale, UMR 1158 BioEcoAgro, Institut Charles Viollette, Unité Sous Contrat ANSES, INRAe, Univ. Artois, Univ. Lille, Univ. de Picardie Jules Verne, Univ. de Liège, Junia, Boulogne-sur-Mer, France

**Keywords:** antimicrobial resistance genes, contamination indicator genes, seawater, qPCR, environmental factors, mobile genetic element

## Abstract

The marine environment is a potential natural reservoir of antimicrobial resistance genes (ARGs), subject to anthropogenic effluents (wastewater, industrial, and domestic), and known as a final receiving system. The aim of this study was to investigate the abundance and geographical distribution of the three *bla_TEM_*, *sul1*, and *intI1* genes, proposed as indicators of contamination to assess the state of antimicrobial resistance in environmental settings, added to the *tetA* gene and the microbial population (*tuf* gene) in the English Channel and North Sea areas. Bacterial DNA was extracted from 36 seawater samples. The abundance of these genes was determined by quantitative PCR (qPCR) and was analyzed in association with environmental variables and geographical locations to determine potential correlations. The *bla_TEM_* and *tetA* genes were quantified in 0% and 2.8% of samples, respectively. The *sul1* and *intI1* genes were detected in 42% and 31% of samples, respectively, with an apparent co-occurrence in 19% of the samples confirmed by a correlation analysis. The absolute abundance of these genes was correlated with the microbial population, with results similar to the relative abundance. We showed that the *sul1* and *intI1* genes were positively correlated with dissolved oxygen and turbidity, while the microbial population was correlated with pH, temperature and salinity in addition to dissolved oxygen and turbidity. The three *tetA*, *sul1*, and *intI1* genes were quantified in the same sample with high abundances, and this sample was collected in the West Netherlands coast (WN) area. For the first time, we have shown the impact of anthropogenic inputs (rivers, man-made offshore structures, and maritime activities) and environmental variables on the occurrence of three indicators of environmental contamination by antimicrobial resistance in the North Sea and English Channel seawaters.

## Introduction

Antimicrobial resistance is an emerging public health threat of the 21st century worldwide. In fact, an incommensurate increase in antimicrobial resistant bacteria (ARB) and antimicrobial resistance genes (ARGs) dissemination in the environment is influencing by the human (hospital, domestic) and animal infection treatments (domestic, animal husbandry, and aquaculture). Other anthropogenic inputs (mainly industrial and agricultural effluents) also threat the water quality with the continuous release of antibiotic residues, ARB and ARGs in aquatic environments. Indeed, water environment is known as a natural reservoir of ARB and ARGs. Many studies have determined the occurrence of ARGs in hospital and domestic effluents (Rodriguez-Mozaz et al., 2015), wastewater treatment plant effluents (Pazda et al., 2019) and aquaculture farm effluents (Su et al., 2020a). These types of effluents converge in rivers and streams (Calero-Cáceres et al., 2017) before being discharged into the marine environment, the final receiving system. The occurrence of ARGs in the marine waters is understudied, or concerns mainly the coastal and estuarine waters which are strongly influenced by various effluents and human activities. For instance, the *bla_TEM_* (β-lactam resistance), *sul1*, *sul2* (sulfonamide resistance), *tetA* and *tetB* (tetracycline resistance) genes have been detected in coastal waters of different countries such as in the coasts of Portugal and England (Alves et al., 2014; Leonard et al., 2018). Once released into the environment, the maintenance and dissemination of ARGs may be influenced by biotic (plants, microbes, and animals) and abiotic factors (amount of oxygen, temperature, pH and salinity; Barkovskii et al., 2012). Only few studies reported the relationships between these factors and the occurrence of ARGs in the aquatic environment. The ARGs can naturally diffuse into aquatic environments *via* water currents, associated with bacteria or as free DNA, then be absorbed by bacteria through transformation or be transferred between bacteria by conjugation and transduction. Therefore, the main mechanisms allowing the dissemination of resistance genes are horizontal gene transfers by mobiles genetic elements (MGEs), like the class 1 integrons which are widely found in the environment (Gillings et al., 2015). The *intI1* gene encoding for the class 1 integron-integrase has been detected in coastal waters in China (Su et al., 2020b) and in urban-impacted coastal estuarine in South Carolina, United States (Uyaguari et al., 2013). Because of the diversity of the ARG types within the environment, it is complicated or even infeasible to detect all the genes constituting the resistome of marine environment. Therefore, some genetic determinants of antimicrobial resistance such as the *bla_TEM_*, *sul1*, and *intI1* genes have been proposed as indicators of contamination to assess the state of antimicrobial resistance in the environment (Berendonk et al., 2015; ANSES, 2020). Furthermore, other genes could be monitored in the marine environment, such as the *tetA* gene encoding tetracycline resistance. Indeed, this gene as well as the indicator genes that have been suggested are clinically relevant, often associated with MGEs and are prevalent in the environment. To our knowledge, no data is available on the occurrence of the *tetA*, *bla_TEM_*, *sul1*, and *intI1* genes in the North Sea and the English Channel seawaters, and no information on the possible role of anthropogenic inputs, and biotic and abiotic factors on this occurrence.

The North Sea and the English Channel areas are distinguished from the vast marine and oceanic environments by more pronounced anthropic influences that have been intensively affected by human activities, especially agriculture and the release of untreated domestic sewage, particularly with the proximity of the English, French, Belgian, Dutch, German, and Danish coasts. In addition, the English Channel and the North Sea are subject to the numerous effluents of the large rivers running through all these countries, such as the Seine (France), the Thames (England) and the Rhine (Netherlands) which contain different natures of pollutants. It is also necessary to highlight the anthropic impact from important European port activities such as Le Havre (France), Rotterdam (Netherlands) and Anvers (Belgium). Similarly, different types and sizes of boats (fishing, container, passenger and cargo ships) discharge the sewage after more or less effective decontamination. In this study, we investigated the absolute abundance and the geographical distribution of the four *bla_TEM_*, *tetA*, *sul1*, and *intI1* indicator genes and the bacterial *tuf* gene to estimate the microbial population in the English Channel and the North Sea seawater samples, using quantitative PCR (qPCR). In order to estimate the proportion of these indicator genes within the microbial population, we also calculated the relative abundance. Finally, we evaluated the influence of environmental variables on the absolute abundance of the indicator genes and the microbial population (Lazard et al., 2021).

## Materials and Methods

### Sample Collection and Sample Preparation by Filtration

**Figure 1:**
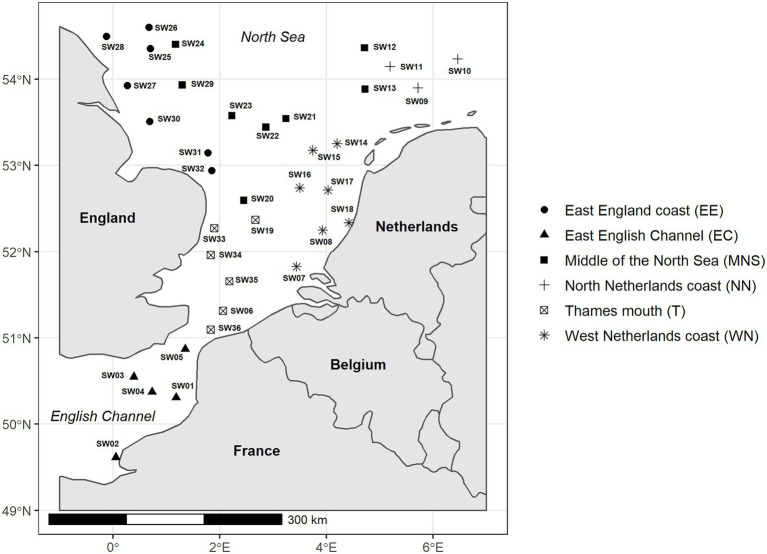
The geographical locations of seawater sampling (SW01–SW36) sites in the English Channel and North Sea, divided into six defined areas.

Seawater samples were collected during the International Bottom Trawl Survey (IBTS) oceanographic campaign in January 2020 in the English Channel—North Sea area (Lazard et al., 2021). Thirty-six seawater samples were collected in six areas defined as East English Channel (EE; *n* = 5), Thames mouth (T; *n* = 6), East England coast (EC; *n* = 7), Middle of the North Sea (MNS; *n* = 8), North Netherlands coast (NN; *n* = 3), and West Netherlands coast (WN; *n* = 7; Figure 1). At each sampling point, 600 ml of seawater were collected at a depth of about 30 m using Niskin bottles and transferred into sterile containers. The samples were stored at −20°C on the boat, then transported to laboratory and stored at −20°C until filtration. Additionally, environmental variables including water temperature, pH, salinity, turbidity, dissolved oxygen and total algae concentration were measured. A request for access to these environmental data can be made to the following address: http://data.ifremer.fr/pdmi/portalssearch/main. Prior to filtration, samples were thawed at room temperature and homogenized. Each seawater sample was successively filtered through 1.2, 0.45, and 0.22 μm pore-size mixed cellulose ester membranes (Millipore, Burlington, United States) using a Nalgene individual vacuum filtration system (Thermo Fisher Scientific, Waltham, United States). Then, the 0.45 and 0.22 μm filter membranes were picked up and sterilely cut into about 1 mm slices. They were transferred in 2 ml tubes and stored at −20°C until DNA extraction.

### Total DNA Extraction

Total DNA was extracted from the 0.45 and 0.22 μm filter membranes using the DNeasy PowerBiofilm kit (Qiagen, Hilden, Germany), following manufacturer’s instructions with some modifications. The membrane slices were placed into bead tubes containing 700 μl of MBL buffer and 200 μl of FB buffer. The tubes were twice vortexed at maximum speed for 45 s and incubated at 56°C for 30 min. Then, we added 2 mg.ml^−1^ of proteinase K (Sigma, Saint-Louis, United States) to the tubes before incubating them at 56°C for 30 min. The tubes were vortexed 15 s and centrifuged at 4,000 × *g* for 1 min. Supernatant was transferred into a 2 ml tube and then centrifuged at 13,000 × *g* for 1 min. Following extraction steps correspond to those described in the extraction kit protocol except of the IRS buffer volume (200 μl) and the final elution step with 2 × 30 μl of EB buffer. The DNA extracted from the two filter membranes were pooled together to form a single DNA extract for the subsequent analysis. The DNA concentration (A260 nm) and purity (A260/280nm and A260/230nm ratios) of each DNA sample were validated using a DS-11 spectrophotometer (Denovix, Wilmington, United States). The DNA extracts were then stored at −20°C until analysis.

### Quantification of the Four *tetA*, *bla_TEM_*, *sul1*, and *intI1* Indicator Genes by qPCR

The indicator genes (*tetA* encoding for tetracycline resistance, *bla_TEM_* for β-lactam resistance, *sul1* for sulfonamide resistance and *intI1* encoding for the class 1 integron-integrase) were quantified by the qPCR method. The different sets of primers and probes used for these qPCR reactions are detailed in the Table 1. Each qPCR was performed in a 20 μl reaction mixture. For the quantification of the *sul1* (Heuer and Smalla, 2007) and *intI1* (Barraud et al., 2010) genes, we employed the qPCR method using TaqMan probes. The mixtures contained 10 μl of LightCycler Probes Master (Roche, Rotkreuz, Switzerland), 0.2 μM of probes (Eurobio, Les Ulis, France), 0.4 μM of each primer (Eurobio) and 5 μl of DNA extract. For the quantification of the *tetA* (Ng et al., 2001) and *bla_TEM_* (Bibbal et al., 2007) genes, we employed the qPCR method using SybrGreen dye. The mixtures contained 10 μl of LightCycler 480 SYBR Green I Master (Roche), 0.4 μM of each primer (Eurobio) and 5 μl of DNA extract. For qPCR positive controls and the carrying out of standard curves, we used three *Escherichia coli* strains containing either the pRP4 plasmid with the *tetA* gene (CIP RP4), pIP69 plasmid with the *bla_TEM,_* gene (CIP pIP69), pR1 plasmid with the *sul1* gene (CRBIP19.58) (Collection of the Institut Pasteur Paris, Paris, France) and the *E. coli* strain containing the pTRC99A plasmid with the *intI1* gene (Barraud et al., 2010). These *E. coli* strains were grown in 10 ml of Trypticase Soy Broth with 0.6% Yeast Extract (TSBYE) containing 100 μg.ml^−1^ of ampicillin (Sigma) and incubated for 24 h at 37°C. After centrifugation for 10 min at 10,000 × *g*, plasmid DNA was extracted from the cell pellets using the QIAprep Spin Miniprep kit (Qiagen) according to the manufacturer’s instructions. For qPCR negative controls, we used three *Vibrio parahaemolyticus* strains (ANSES B3PA Collection, Boulogne-sur-Mer, France): B3PA-12-1,629 strain without *bla_TEM_* gene, B3PA-13-2,931 strain without the *intI1* gene and B3PA-15-0205 strain without the *tetA* and *sul1* genes. These *Vibrio* strains were isolated on saline nutrient agar and incubated for 24 h at 37°C, before DNA extraction using DNeasy Blood and Tissue kit (Qiagen) following manufacturer’s instructions. All qPCR reactions (TaqMan probe and SybrGreen dye) were performed according to the conditions detailed in the Table 1 using a LightCycler 480 thermal cycler (Roche). Quantification cycle (Cq) values were calculated using the second derivate method.

**Table 1:** Primer and probe sequences and conditions associated to the quantitative PCR (qPCR) used in this study.

Gene	Primer/probe sequences	qPCR conditions	qPCR parameters	References
*tuf*	ACHGGHRTHGARATGTTCCG GTTDTCRCCHGGCATNACCAT	95°C—5 min (1 cycle); 95°C—10 s, 60°C—30 s, 72°C—15 s (45 cycles)	*E* = 73.4% *r*^2^ = 0.9989	Tanaka et al., 2010
*tetA*	GCTACATCCTGCTTGCCTTC CATAGATCGCCGTGAAGAGG	95°C—5 min (1 cycle); 95°C—10 s, 60°C—30 s, 72°C—5 s (45 cycles)	*E* = 84.8% *r*^2^ = 0.9873	Ng et al., 2001
*bla_TEM_*	TTCCTGTTTTTGCTCACCCAG CTCAAGGATCTTACCGCTGTTG	95°C—5 min (1 cycle); 95°C—10 s, 60°C—30 s, 72°C—5 s (45 cycles)	*E* = 93.5% *r*^2^ = 0.9992	Bibbal et al., 2007
*intI1*	GCCTTGATGTTACCCGAGAG GATCGGTCGAATGCGTGT (6-FAM)ATTCCTGGCCGTGGTTCTGGGTTTT(BHQ1)	95°C—5 min (1 cycle); 95°C—10 s, 60°C—1 min, 72°C—1 s (45 cycles)	*E* = 103.3% *r*^2^ = 0.9953	Barraud et al., 2010
*sul1*	CCGTTGGCCTTCCTGTAAAG TTGCCGATCGCGTGAAGT (FAM)CAGCGAGCCTTGCGGCGG(TAMRA)	95°C—5 min (1 cycle); 95°C—10 s, 60°C—1 min, 72°C—1 s (45 cycles)	*E* = 110.1% *r*^2^ = 0.994	Heuer and Smalla, 2007

### Quantification of Microbial Population (*tuf* Gene) by qPCR

The quantification of the bacterial housekeeping *tuf* gene (Tanaka et al., 2010) was performed by qPCR method using SybrGreen dye to assess the relative abundance of indicator genes in relation to the microbial population. The primers are detailed in the Table 1. The reaction mixture of 20 μl contained 10 μl of LightCycler 480 SYBR Green I Master (Roche), 0.4 μM of each primer (Eurobio) and 2 μl of DNA extract. Nuclease-free water was added to complete the total reaction volumes and was used for the negative control. For qPCR positive control and for the standard curve, we used the *V. parahaemolyticus* B3PA-16-0006 strain (ANSES B3PA Collection; Briet et al., 2018). Briefly, this strain was isolated on saline nutrient agar and incubated for 24 h at 37°C before DNA extraction using DNeasy Blood and Tissue kit (Qiagen) following manufacturer’s instructions. All qPCR reactions were performed according to the conditions detailed in the Table 1 using a LightCycler 480 thermal cycler (Roche). Cq values were calculated using the second derivate method.

### Data Analysis and Statistics

The qPCR results were validated following these criteria: the efficiency ranged from 75% to 125% (according to the NF T90-471:2015-06 standard), the linearity *r*^2^ value was ≥0.99 and the Cq values of the negative controls were below the threshold of sensitivity. Gene copy numbers were calculated by the standard curves. The sensitivity for the qPCR targeting the *tetA* and *bla_TEM_* genes was set at 10^5^ copies.ml^−1^ while it was set at 10^4^ copies.ml^−1^ for the *sul1*, *intI1*, and *tuf* genes. The absolute abundance was the concentration of the genes per mL of seawater (log copies.ml^−1^ seawater). Then, to calculate the relative abundance of the indicator genes (*tetA*, *bla_TEM_*, *sul1*, and *intI1* genes), we normalized the absolute abundance of the indicator genes with the absolute abundance of the *tuf* gene (Chen et al., 2019). All data presentation and statistical analysis were performed using RStudio Software version 1.4.1717 (RStudio, Inc., Boston, United States). One-way ANOVA and Tukey’s multiple pairwise-comparisons were used to assess statistically significant differences at the 95% CI (*p* < 0.05). These analyses were performed on the absolute and relative abundances results to assess the dissemination of the genes according to the geographical areas. Possible correlations between the absolute abundances of the genes and the environmental variables were determined with Pearson’s rank correlation coefficient (*r*). The power of the correlations was defined as follows: very low correlation (0 < *r* < 0.19), low correlation (0.20 < *r* < 0.39), moderate correlation (0.40 < *r* < 0.59), high correlation (0.60 < *r* < 0.79), very high correlation (0.80 < *r* < 0.99), and perfect correlation (*r* = 1.0). The correlation value of *p* were produced with a significance test to assess the relationships between the targeted genes and the environmental variables.

## Results

### Absolute Abundance of the Four *tetA*, *bla_TEM_*, *sul1*, and *intI1* Indicator Genes and the *tuf* Gene

**Figure 2:**
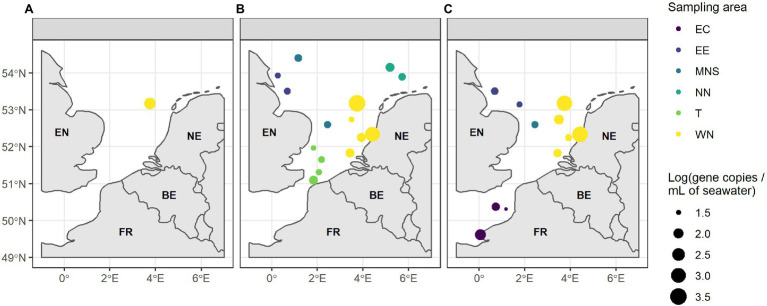
Absolute abundance of the three **(A)** *tetA*; **(B)** *sul1*, and **(C)** *intI1* indicator genes (gene copies.ml^-1^ of seawater, log-transformed) in the six areas. EC, East English Channel; EE, East England coast; MNS, Middle of the North Sea; NN, North Netherlands coast; T, Thames mouth; and WN, West Netherlands coast. EN, England; NE, Netherlands; FR, France; and BE, Belgium. The samples with no quantification of genes are not shown.

**Figure 3:**
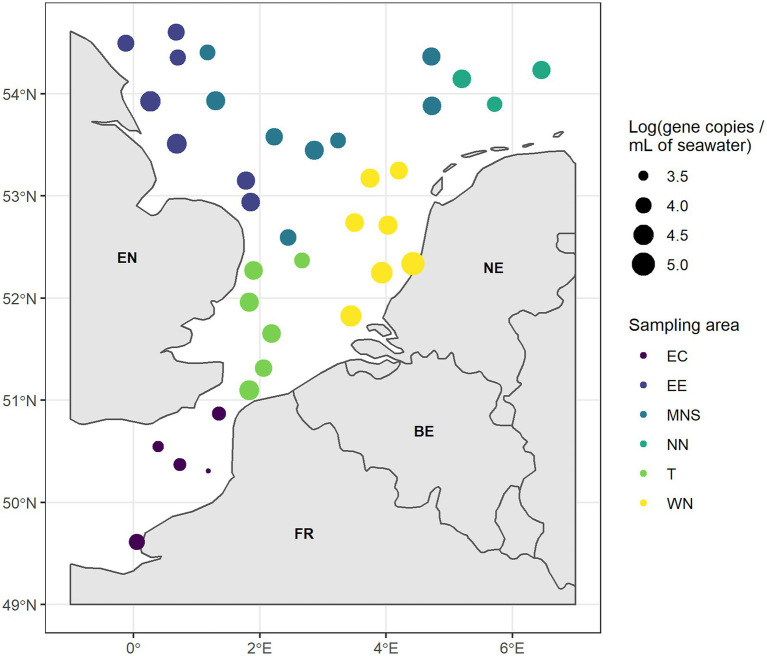
Absolute abundance of the *tuf* gene (gene copies.ml^-1^ of seawater, log-transformed) in the six areas. EC, East English Channel; EE, East England coast; MNS, Middle of the North Sea; NN, North Netherlands coast; T, Thames mouth; and WN, West Netherlands coast. EN, England; NE, Netherlands; FR, France; and BE, Belgium.

The absolute abundance of the four indicator genes and the *tuf* gene in the seawater samples and their geographical distribution are shown in Figures 2, 3. The absolute abundance values of the indicator genes and the *tuf* gene are referenced in the Supplementary Table S1. The *bla_TEM_* gene was not quantified in any seawater sample. However, we quantified the *tetA* gene in only one out of the 36 analyzed seawater samples, with an absolute abundance of 2.24 log copies.ml^−1^ of seawater in the WN area (Figure 2A). The *sul1* gene was quantified in 15/36 analyzed seawater samples with absolute abundances ranging from 1.52 to 3.55 log copies.ml^−1^ of seawater (Figure 2B). We observed a lower absolute abundance in four areas (EE, MNS, NN, and the T areas) compared to the WN area. Moreover, it was not quantified in the East English Channel area (EC). For the *intI1* gene, we found it in 11/36 analyzed seawater samples (Figure 2C). This gene was quantified neither in the T nor in the NN areas. We observed an absolute abundance of approximately 1.60 log copies.ml^−1^ of seawater in two samples collected near the EE and in one sample from the MNS area. The *intI1* gene absolute abundance was higher in the EC area (2.16 log copies.ml^−1^in one sample) and in the WN area, with two samples showing absolute abundances of 3.14 and 3.26 log copies.ml^−1^ of seawater. The *tuf* gene was quantified in all seawater samples, with absolute abundance ranging from 3.38 to 5.02 log copies.ml^−1^ of seawater (Figure 3). We observed significantly higher absolute abundance in the WN area (between 1.60 and 3.26 log copies.ml^−1^) compared to the EC (1.47 to 2.16 log copies.ml^−1^) (*p* = 0.007) and to the MNS (1.64 log copies.ml^−1^) (*p* = 0.050) areas.

### Relative Abundance of the Indicator Genes

**Figure 4:**
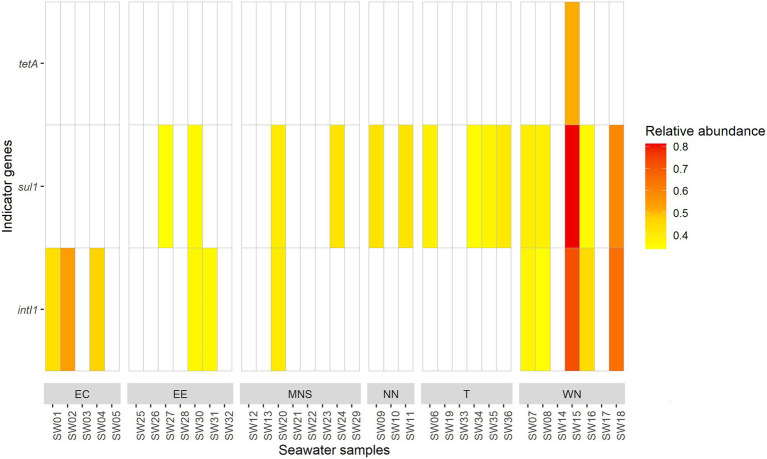
Relative abundance of the *tetA*, *sul1*, and *intI1* indicator genes in the seawater samples, for the six areas. EC, East English Channel; EE, East England coast; MNS, Middle of the North Sea; NN, North Netherlands coast; T, Thames mouth; and WN, West Netherlands coast. The color key indicates the relative abundance in the respective samples. The white rectangles indicate an absence of quantification. The *bla_TEM_* gene was not quantified.

The relative abundance of the *tetA* gene in the SW15 sample was 0.51 (Figure 4). For the *sul1* gene, we observed relative abundance ranging from 0.34 to 0.81. In the EE coast, MNS, NN, and T areas, constant relative abundances (around 0.40) were observed. The relative abundances were higher in the SW15 (0.81) and SW18 samples (0.60) collected in the WN area. For the *intI1* gene, the relative abundance ranged from 0.34 to 0.72. The relative abundance of the *intI1* gene was significantly higher in the WN area compared to the MNS (*p* = 0.040) area. Indeed, two samples (SW15 and SW18) in the WN area had higher relative abundance compared to the other analyzed samples in the same area and in the two other areas. The relative abundance results of the *sul1* and *intI1* genes were in agreement with those of absolute abundances. Moreover, no quantification of genes was observed in 17/36 (47.22%) seawater samples. When only one gene (*sul1* or *intI1* gene) has been detected (33.33% seawater samples), we found exclusively the *intI1* gene in the East English Channel (EC) area and the *sul1* gene in the NN and T areas. Among the seven samples with co-occurrence of the *sul1* and *intI1* genes, the higher relative abundances were observed in two seawater samples (SW15 and SW18 samples in the WN area). Furthermore, the highest relative abundance of the three *tetA*, *sul1*, and *intI1* genes concerned the same sample (SW15 sample), collected in the WN area.

### Correlations Between the Absolute Abundance of the *sul1*, *intI1*, *tuf* Genes and the Environmental Variables

**Figure 5:**
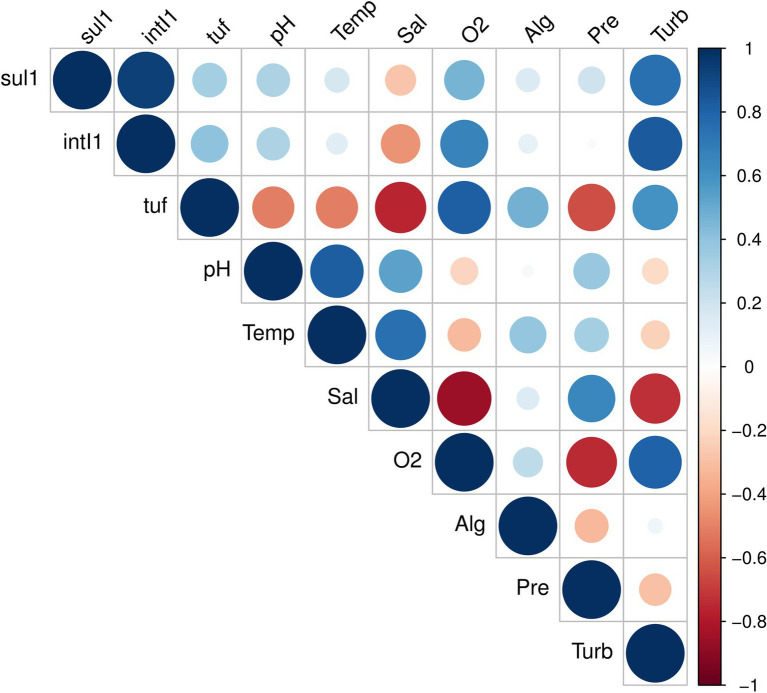
Pearson correlation matrix between the sul1 and intI1 indicator genes, the tuf gene and the environmental variables. Temp, temperature; Sal, salinity; O_2_, dissolved oxygen; Alg, algal biomass; Pre, pressure; and Turb, turbidity. The color key indicates the correlation coefficients and corresponding colors.

Since the *bla_TEM_* gene was not quantified and the *tetA* gene in only 2.78% of the seawater samples, their abundance was not considered in the analysis of correlations with environmental variables. In the analyzed seawater samples, the absolute abundance of the *intI1* gene was strongly and positively correlated with the *sul1* gene (*r* = 0.94, *p* = 0.000; Figure 5). Moreover, the absolute abundance of the *intI1* gene was positively and highly correlated with the dissolved oxygen concentration (*r* = 0.66, *p* = 0.003) and very highly with the turbidity values (*r* = 0.84, *p* = 0.000). We also observed a moderate correlation between the *sul1* gene abundance and the dissolved oxygen concentration (*r* = 0.46, *p* = 0.030) and a high correlation between this gene and the turbidity values (*r* = 0.75, *p* = 0.001). A low positive correlation was observed between the absolute abundance of these genes and the pH values (*r* values of approximately 0.30). In contrast, the absolute abundances of the *sul1* and *intI1* genes were low and moderately negatively correlated with salinity values, with *r* values of −0.27 and −0.44 and value of *p* 0.030 and 0.005, respectively. Furthermore, we observed low positive correlation between these two genes and the temperature, algal biomass and pressure values, with r coefficients ranging from 0.02 to 0.21. Regarding the *tuf* gene, which estimates the bacterial concentration in the seawater samples, we observed a very high positive correlation with the dissolved oxygen concentration (*r* = 0.81, *p* = 0.000) and moderate correlations with the turbidity (*r* = 0.59, *p* = 0.001) and the algal biomass values (*r* = 0.48). Moderate negative correlations were observed with the pH (*r* = −0.51, *p* = 0.001) and temperature values (*r* = −0.51, *p* = 0.000), and highly negative correlations with salinity (*r* = −0.76, *p* = 0.000) and pressure values (*r* = −0.65, *p* = 0.000). In addition, the *tuf* gene abundance was slightly and positively correlated with the abundance of the *sul1* and *intI1* genes (*r* = 0.34 and 0.40, respectively).

## Discussion

This study provided for the first time information on the presence of indicator genes of antimicrobial resistance contamination in the North Sea and English Channel seawaters. We detected at least one of the indicator gene in 52.78% of the samples, two indicator genes in 19.44% of the samples and three indicator genes in 2.78% of the samples analyzed. In contrast, the four indicator genes were not detected in a same seawater sample. The *bla_TEM_* gene encoding resistance to β-lactam was not detected in any sample, and the *tetA* gene encoding resistance to tetracycline was quantified in only one sample. Their presence in the marine environment, in *E. coli* strains isolated from coastal marine waters, has been demonstrated in Portugal (Alves et al., 2014). In this study, it is possible that the two *tetA* and *bla_TEM_* genes were not present or present below the limit of quantification because the marine environment is a diluted medium. The filtration of a larger volume of seawater could confirm or not this hypothesis. In addition, gene quantification in environmental matrix such as seawater may be affected by the presence of PCR inhibitors. Inhibitory substances such as polyphenols, humic and fumic acids or glycogen have been isolated from the marine environment and are known to be one of the most common causes of qPCR failure (Schrader et al., 2012). In contrast, the *sul1* and *intI1* genes were the most abundant in this study, in 42% and 31% of the seawater samples, respectively. Co-occurrence of these two genes was observed in 19% of the samples, and the correlation analysis carried out also illustrated a strong correlation between the *sul1* and *intI1* genes. The *sul1* gene was present alone in 53% of the samples (without *intI1*), and by contrast, *intI1* was present in 36% of the samples without the *sul1* gene. These observations were also reported in multidrug resistant isolates from river systems in India, where 72.6% of the strains had at least one integron with the *sul1* gene and 8.26% had the *sul1* gene but not the *intI1* gene (Chaturvedi et al., 2021). Indeed, class 1 integrons are composed of a 5′ conserved region with the *intI1* gene and a 3′ conserved region with a sulfonamide resistance gene (such as *sul1*), between which one or more ARG-containing cassettes can be inserted. But other genes encoding for sulfonamide resistance can be identified instead of *sul1* within these structures, such as the *sul2* and *sul3* genes (Jones-Dias et al., 2016), which could explain the high prevalence of the *sul1* gene without *intI1*. The integrons are mainly found in Gram-negative bacteria (such as *Enterobacteriaceae*, *Pseudomonas*, *Acinetobacter*, *Vibrio*, and *Campylobacter*), whether they are environmental or clinical isolates and may carry acquired resistance genes, thus constituting a human health risk. The abundance of the *sul1* and *intI1* genes was highly correlated with the dissolved oxygen concentration and turbidity, which exhibits suspended particulate matters such as microorganisms or particles in suspension where microorganisms aggregate. Similarly, Tao et al. (2016) showed that dissolved oxygen could be an important factor in ARGs selection in wastewater treatment systems, affecting the microbial communities that may be carriers of these resistances. A recent study on the occurrence of ARGs in an estuary in China also showed a correlation between turbidity and ARGs, with a higher prevalence of these genes in areas with high turbidity (Chen et al., 2020). To assess the contribution of the microbial population in the dissemination of ARGs in the marine environment, we quantified the bacterial housekeeping *tuf* gene to estimate the concentration of bacteria and to calculate the relative abundance of indicator genes. Indeed, we targeted the *tuf* gene rather than the 16S rRNA gene, since it has been shown that the 16S rRNA gene has similarities with the 18S eukaryotic mitochondria gene, which can therefore interfere with the quantification of bacterial DNA in complex samples (Nakano, 2018). The analysis of the correlation between the *sul1*, *intI1* genes, and the bacterial *tuf* gene showed a slight correlation between the abundance of the two contamination indicators and the estimated microbial population. This may demonstrate that the indicator genes quantified in this study are not only found in bacteria targeted by the *tuf* gene, but also as free DNA in water or in association with bacteriophages, as has already been shown (Calero-Cáceres and Balcázar, 2019; Sivalingam et al., 2020). This assumption was corroborated with the relative abundance results, which were similar to the absolute abundances. We demonstrated a strong correlation between the microbial population and dissolved oxygen, which could reflect the presence of bacteria that thrive in aerobic environments such as close to the sea surface. As well, we demonstrated a correlation between the microbial population and the turbidity. We have observed that slight pressure and pH variations in seawater did not affect the abundance of the *sul1* and *intI1* genes, but only the microbial population. Currently, it has been shown that pH did not influence the occurrence of ARGs in seawater but only in marine sediments (Chen et al., 2020) and in river sediments (Ergie et al., 2019). Other abiotic factors in the environment can also exert selection pressure, playing a role in the maintenance and proliferation of antimicrobial resistances. Indeed, it has been demonstrated that environmental bacteria can be exposed to selection pressure by antibiotic residues (natural origin or brought by various anthropogenic sources), and thus develop resistance (Larsson, 2014). The occurrence of indicator genes in seawater samples diverged according to the geographical area considered, i.e., the type of anthropogenic impact but, given the limited data on English Channel and North Sea areas, several hypotheses can be advanced considering the geographical location of the samples. High abundance of the three *tetA*, *sul1*, and *inti1* genes was noticed in the West of the Netherlands. Certain factors could explain this abundance such as human wastewater originating from the man-made structures in this area and the animals (fish, mammals, and seabirds) contaminated by ARGs and ARB. These man-made structures refer to the diverse offshore installations present in the North Sea, including platforms for gas and oil exploitation. The various human discharges from these structures could constitute an additional impact on the dissemination of ARGs in the marine environment, but to our knowledge, no study has been conducted on the wastewater decontamination treatment efficiency of these structures for the removal of biological contaminants such as ARB and ARGs. High abundance of the *sul1* and *intI1* genes (approximately 3 log copies.ml^−1^) was also observed slightly further south, where the North Sea receives river water from the North Sea Canal. This canal is subject to various anthropogenic discharges, notably from Amsterdam and its port. It is now recognized that river waters are contaminated with various pollutants, including ARGs, which are not fully removed in wastewater treatment plants and are disseminated in the downstream environment (Zhang et al., 2009). Furthermore, three offshore wind farms are also visible in this area not far from the coast, potentially constituting an additional impact on the presence of ARGs. Indeed, more than 120 offshore wind platforms have been constructed in Europe over the last years, the majority of which are in the North Sea (WindEurope, 2019). A study in the North Sea has shown that offshore wind farms attract certain bird species, such as black-backed and herring gulls (Vanermen et al., 2014), and gull feces have been consistently shown to be a source of ARG contamination. In Portugal, *E. coli* strains carrying different *bla*, *sul*, and *tet* genes have been isolated from seagull feces (Poeta et al., 2008). This reveals a potential involvement of contaminated gull feces in the pollution of marine environment by ARGs. The *sul1* and *intI1* genes were also found even further south, near the mouths of the Rhine and Meuse rivers, two large rivers flowing into the North Sea. In a recent study, these two genes were quantified along the entire length of the Rhine in Switzerland, Germany and the Netherlands at different concentrations (Paulus et al., 2020). The authors showed that the presence and concentration of ARGs and *intI1* fluctuated greatly between different sampling points, but that the concentration of the *sul1* and *intI1* genes was highest in two areas polluted by well-developed pharmaceutical industries and downstream, in Lower Rhine. The impact of these river effluents on pollution in the North Sea is cumulative with the impact of the city and the port of Rotterdam, the largest seaport in Europe. The port of Rotterdam is characterized by a high maritime traffic (more than 30,000 ships call at this port each year) and by large industrial areas composed of oil refineries and chemical industries, but there is no information about the contamination by ARGs of port activity effluents. In the East of England, the *sul1* and *intI1* genes were quantified in the same seawater sample, collected south of the Humber estuary. This marine estuary is the largest source of freshwater that England discharges into the North Sea. A study in these estuarine waters identified *V. parahaemolyticus* strains resistant to various antibiotics (kanamycin, gentamicin, cefazolin, tetracycline; Daramola et al., 2009), indicating a potential source of ARGs in this estuary. In addition, this area includes several nearby wind farms, as well as various ports upstream of the Humber Estuary (Port of Hull, Green Port Hull, Immingham). Finally, the *sul1* and *intI1* genes were poorly quantified in the MNS (2/8 and 1/8 samples from this area, respectively). This area, unlike the others, is quite far from the coasts and thus from their influence, but has a concentration of offshore platforms for gas and oil exploitation (Fujii, 2015). However, we have quantified the *sul1* and *intI1* genes in seawater samples collected far from the coast, in areas that could be described as subject to little anthropogenic impact. This spreading of genes within the marine environment may raise questions about the involvement of intense maritime traffic and their various discharges, such as ballast water, which is pumped out and discharged into the sea. Few studies have been conducted on the contamination of these ballast waters by ARB and ARGs, and their dissemination in marine waters, but it has been estimated that approximately 10^19^ bacteria are transported worldwide each day in ship ballast water (Brinkmeyer, 2016). In addition, a recent study demonstrated the presence of the *sul1* and *intI1* genes at high concentrations (between 10^3^ and 10^8^ gene copies per 100 ml of water) in ballast waters in two ports in China, from ships coming from all over the world (Australia, Japan, Europe, Korea, Asia, South America; Lv et al., 2020). The replacement of water in tanks occurs at the shoreline, but also in open seawaters, potentially creating long-range dispersion of contaminants. In addition to this ballast waters, we must also consider the impact of wastewater discharges from boats, such as black water (wastewater from bathrooms and toilets) and gray water (wastewater from sinks, washing machines, and bathtubs). Traces of antibiotic residues have been detected in these types of water, such as sulfamethoxazole (from the sulfonamide family), in cruise ships in the port of Hamburg, Germany (Westhof et al., 2016), which can exert a selection pressure on the surrounding bacteria and induce the appearance of antimicrobial resistant strains. In the English Channel, only the *intI1* gene has been quantified. In this area, we observed a higher abundance in the sample collected near Le Havre, the second-largest port in France for maritime traffic and the fifth-largest European port for container traffic, located at the Seine mouth. A study has also exhibited the presence of the *intI1* gene in this river, which is the final destination of effluents from 16 wastewater treatment plants (Laroche et al., 2009). This gene was detected in *E. coli* isolates all along the Seine (France), with a higher prevalence in the mouth of the estuary near Tancarville (France) and Honfleur (France), two cities located just next to Le Havre (France). The English Channel is considered a “highway of the sea,” with around 800 boats sailing through these waters every day (Amara, 2010). By contrast, a little further north at the mouth of the Thames (England) and in northern France, only the *sul1* gene has been quantified. This area is subject to discharges from the Thames (England), a 346 km long river that is itself subject to numerous effluents. Several studies have reported that these waters are polluted, particularly by antibiotic residues and ARGs. For example, White et al. (2019) detected three sulfonamide antibiotics (sulfadiazine, sulfamethoxazole, and sulfanilamide) along the Thames River (England) up to the mouth after seven wastewater treatment plants, potentially exerting selection pressure on bacteria and leading to sulfonamide-resistant strains. In addition, the *bla_TEM_*, *tetA*, *sul1*, and *intI1* genes were quantified in these waters at 10^6^–10^8^ copies.L^−1^ of water (Xu et al., 2019). The sample with the highest *sul1* gene abundance in this area was collected near the French coast, around Calais. This area is subject to the passage of many passenger ships, which connect the city of Calais (France) to Dover (England). The occurrence of the *sul1* gene has also been demonstrated in the north of the Netherlands, away from the coast, distinguished by the presence of an offshore wind farm. All of these man-made constructions (oil and gas platforms, offshore wind farms) and effluents (rivers, streams, sewage and ballast water from ships) are therefore potential sources of contamination of marine waters by ARGs.

## Conclusion

We have shown for the first time that the seawaters of the English Channel and the North Sea were contaminated with ARGs and MGEs. The marine environment is very complex to study, due to many potential sources of contamination such as different effluents, intense maritime traffic and man-made structures in addition to the impact of environmental conditions on their prevalence and maintenance in the environment, which have been understudied. We believed that the *sul1* and *intI1* genes would be suitable contamination indicators to assess the state of antimicrobial resistance in marine environment, but that other ARGs should be monitored in conjunction with these two genes, such as the *tetM* and *bla_CTX-M_* genes, also proposed as indicator genes by Berendonk et al. (2015). In addition to seawater analysis, other matrices should be considered such as plankton, fish and bivalve mollusks to characterize the transmission and dissemination of ARGs along marine food webs up to us, final consumers. Indeed, the monitoring of antimicrobial resistance in the marine environment is an important issue, representing a risk to human health, especially through the consumption of fishery products contaminated by antimicrobial-resistant pathogenic bacteria, which can lead to therapeutic failures in case of infection.

## Data Availability Statement

The original contributions presented in the study are included in the article/Supplementary Material; further inquiries can be directed to the corresponding author.

## Author Contributions

EB designed the study, performed the analysis and interpretation of the results, and drafted the manuscript. DC participated in the design of the study, performed the experiments, and contributed to the manuscript writing. TB participated in the design of the study and contributed to the manuscript writing. CB contributed to the manuscript writing. GM participated in the design of the study and contributed to the manuscript writing. All authors contributed to the article and approved the submitted version.

## Funding

This study was supported by a doctoral fellowship from Région Hauts-de-France and Pôle Métropolitain de la Côte d’Opale. The Structure Fédérative de Recherche (SFR) Campus de la Mer has also financially supported this work.

## Conflict of Interest

The authors declare that the research was conducted in the absence of any commercial or financial relationships that could be construed as a potential conflict of interest.

## Publisher’s Note

All claims expressed in this article are solely those of the authors and do not necessarily represent those of their affiliated organizations, or those of the publisher, the editors and the reviewers. Any product that may be evaluated in this article, or claim that may be made by its manufacturer, is not guaranteed or endorsed by the publisher.
