# Supplementary material for: Occurrence of Indicator Genes of Antimicrobial Resistance Contamination in the English Channel and North Sea Sectors and Interactions With Environmental Variables
Source: Front Microbiol. 2022 May 16;13:883081. doi: 10.3389/fmicb.2022.883081 (PMC9150721; doi:10.3389/fmicb.2022.883081)
Supplement: Supplementary file 1 [file Table_1.docx]

|  |  | **Gene absolute abundance (log gene copies.mL^-1^ of seawater)** | | | | |
| --- | --- | --- | --- | --- | --- | --- |
| **Sample** | **Sampling area** | ***bla_TEM_*** | ***tetA*** | ***sul1*** | ***intI1*** | ***tuf*** |
| SW01 | EC | - | - | - | 4.47 | 6.16 |
| SW02 | EC | - | - | - | 5.16 | 6.76 |
| SW03 | EC | - | - | - | - | 6.34 |
| SW04 | EC | - | - | - | 4.74 | 6.45 |
| SW05 | EC | - | - | - | - | 6.56 |
| SW06 | T | - | - | 4.57 | - | 6.90 |
| SW07 | WN | - | - | 4.83 | 4.72 | 7.43 |
| SW08 | WN | - | - | 4.81 | 4.60 | 7.46 |
| SW09 | NN | - | - | 4.67 | - | 6.70 |
| SW10 | NN | - | - | - | - | 6.99 |
| SW11 | NN | - | - | 4.81 | - | 7.07 |
| SW12 | MNS | - | - | - | - | 7.02 |
| SW13 | MNS | - | - | - | - | 7.08 |
| SW14 | WN | - | - | - | - | 6.98 |
| SW15 | WN | - | 2.24 | 6.54 | 6.14 | 7.14 |
| SW16 | WN | - | - | 4.53 | 4.92 | 7.05 |
| SW17 | WN | - | - | - | - | 7.11 |
| SW18 | WN | - | - | 5.99 | 6.26 | 7.79 |
| SW19 | T | - | - | - | - | 6.71 |
| SW20 | MNS | - | - | 4.63 | 4.64 | 6.83 |
| SW21 | MNS | - | - | - | - | 6.71 |
| SW22 | MNS | - | - | - | - | 7.14 |
| SW23 | MNS | - | - | - | - | 6.90 |
| SW24 | MNS | - | - | 4.68 | - | 6.74 |
| SW25 | EE | - | - | - | - | 6.78 |
| SW26 | EE | - | - | - | - | 6.88 |
| SW27 | EE | - | - | 4.54 | - | 7.31 |
| SW28 | EE | - | - | - | - | 6.87 |
| SW29 | MNS | - | - | - | - | 7.10 |
| SW30 | EE | - | - | 4.60 | 4.65 | 7.26 |
| SW31 | EE | - | - | - | 4.55 | 7.02 |
| SW32 | EE | - | - | - | - | 7.05 |
| SW33 | T | - | - | - | - | 7.09 |
| SW34 | T | - | - | 4.52 | - | 7.14 |
| SW35 | T | - | - | 4.61 | - | 7.08 |
| SW36 | T | - | - | 4.82 | - | 7.26 |

**Table S1**: Absolute abundance values of indicator genes and *tuf* gene per seawater sample (SW) (log gene copies.mL^-1^ of seawater). (-): no quantification of the gene. EC: East English Channel; EE: East England coast; MNS: Middle of the North Sea; NN: North Netherlands coast; T: Thames mouth; WN: West Netherlands coast.
